# Estimation of Calcium Titanate or Erbium Oxide Nanoparticles Induced Cytotoxicity and Genotoxicity in Normal HSF Cells

**DOI:** 10.1007/s12011-022-03354-9

**Published:** 2022-07-30

**Authors:** Hanan R. H. Mohamed, Maria M. H. Ibrahim, Esraa S. M. Soliman, Gehan Safwat, Ayman Diab

**Affiliations:** 1 Zoology Department, Faculty of Science, Cairo University, Giza, Egypt; 2 Faculty of Biotechnology, October University for Modern Sciences and Arts, 6Th of October City, Egypt

**Keywords:** Calcium titanate nanoparticles, Erbium oxide nanoparticles, Cytotoxicity, Genotoxicity, Apoptosis, ROS generation

## Abstract

Extensive uses of calcium titanate nanoparticles (CaTiO_3_-NPs) and erbium oxide nanoparticles (Er_2_O_3_-NPs) increase their release into the environment and human exposure, particularly through skin contact. However, there are almost no studies available on the effect of these nanoparticles on skin integrity. Therefore, this study was undertaken to estimate CaTiO_3_-NP- or Er_2_O_3_-NP-induced cytotoxicity and genotoxicity in normal human skin fibroblast (HSF) cells. Cell viability was measured using sulforhodamine B (SRB) assay, while the level of DNA damage was detected using the alkaline comet assay. The intracellular levels of reactive oxygen species (ROS) as well as the expression level of p53, Bax, and Bcl2 genes were detected. Although the viability of HSF cells was non-markedly changed after 24 h, prolonged treatment with CaTiO3-NPs or Er2O3-NPs for 72 h induced concentration-dependent death of HSF cells. Treatment of normal HSF cells with IC50/72 h of CaTiO3-NPs or Er2O3-NPs did not cause marked changes in the intracellular level of ROS, DNA damage parameters, and expression levels of apoptosis genes compared to their values in the untreated HSF cells. We thus concluded that CaTiO3-NPs or Er2O3-NPs cause time- and concentration-dependent cytotoxicity toward normal HSF cells. However, safe and non-genotoxic effects were demonstrated by the apparent non-significant changes in intracellular ROS level, DNA integrity, and apoptotic genes’ expression after exposure of normal HSF cells to nanoparticles. Thus, it is recommended that further studies be conducted to further understand the toxic and biological effects of CaTiO_3_-NPs and Er2O3-NPs.

## Introduction

Nanomaterials represent the most advanced level of nanotechnology in both commercial applications and scientific knowledge due to their unique size-dependent physical and chemical properties [1, 2]. The distinct chemical, physical, mechanical, and optical properties of nanoparticles are gaining impetus for their use in various industrial, biological, and medical applications. For example, nanoparticles are used as drug and delivery formulations, in tissue engineering and tumor destruction by hyperthermia, as probes for DNA structure, and as biosensors [3–5].

Unfortunately, rapid progress in the manufacture and uses of nanoparticles are increasing human exposure to many manufactured nanoparticles such as erbium oxide nanoparticles (Er_2_O_3_-NPs) and calcium titanate nanoparticles (CaTiO_3_-NPs) [6]. The excellent size-dependent electrical and optical properties of Er_2_O_3_-NPs increase their use in many industrial and medical applications. For example, Er_2_O_3_-NPs are used as coloring agent and are also used in the manufacture of displays, in green chemistry synthesis of photoluminescence nanoparticles, and in bio-imaging [7–11].

Likewise, the uniquely attractive physical and chemical properties of CaTiO_3_-NPs are increasing attention in their use in various applications such as environmental processing, catalytic, electronics and photocatalytic processes, biomimetic calcium phosphate formation, and biomedical engineering [12]. Indeed, the unique properties of CaTiO_3_-NPs including high bonding affinity with calcium ions, high surface area to mass ratio, and high chemical and catalytic reactivity increase their uses in many consumer products, paints, and pharmaceuticals, as well as in wastewater treatment [13–15].

Nanoparticles are small enough to penetrate cell membranes and interfere with cellular macromolecules and many subcellular mechanisms [15–17]. However, the impact of Er_2_O_3_-NPs and CaTiO_3_-NPs on cell integrity and genomic DNA has not been well studied. Therefore, this study was conducted to estimate Er_2_O_3_-NP- and CaTiO_3_-NP-induced cytotoxicity and genotoxicity on a normal human skin fibroblast cell line. The sulforhodamine B assay was conducted to assess cell viability and the alkaline comet assay was done to measure DNA damage. Intracellular reactive oxygen species production was detected using 2,7-dichlorofluorescein diacetate dye, and the expression levels of the apoptotic (p53 and Bax) and anti-apoptotic (Bcl2) genes were quantified using real-time polymerase chain reaction.

## Materials and Methods

### Chemicals

Calcium titanate nanoparticles (CaTiO_3_-NPs) and erbium (III) oxide nanoparticles (Er_2_O_3_-NPs) were purchased from Sigma-Aldrich Chemical Company (St. Louis, USA). CaTiO_3_-NPs have a white appearance with product number 633801 and purity of 99% trace metal basis, while Er_2_O_3_-NPs have a pink appearance with product number 203238 and purity of 99.9% trace metal basis. Powders of CaTiO_3_-NPs and Er_2_O_3_-NPs were suspended in deionized distilled water and ultra-sonicated to prepare the concentrations required for the experiments of this study.

### Cell Lines

Normal human skin fibroblasts (HSF) were purchased from Nawah Scientific Company (Mokattam, Cairo, Egypt) and were preserved in Dulbecco’s modified Eagle medium (DMEM) provided with streptomycin (100 mg/mL), penicillin (100 units/mL), and heat-inactivated fetal bovine serum in a humidified, 5% (v/v) carbon dioxide atmosphere at 37 °C.

### Characterization of Nanoparticles

X-ray diffraction (XRD) analysis was conducted to study the purity of CaTiO_3_-NPs and Er_2_O_3_-NPs using a charge coupled device diffractometer (XPERT-PRO, PANalytical, Netherlands). Suspended Er_2_O_3_-NPs and CaTiO_3_-NPs were also imaged using transmission electron microscopy (TEM) to determine the morphology and mean particle size of CaTiO_3_-NPs.

### Cell Viability

The effect of CaTiO_3_-NPs or Er_2_O_3_-NPs on the HSF cell viability was assessed using a sulforhodamine B (SRB) assay [18]. A 100 µL of HSF cell suspensions was cultured separately in 96-well plates and incubated for 24 h in complete media. After incubation, the cells were treated with five different concentrations of CaTiO_3_-NPs or Er_2_O_3_-NPs separately, i.e., 0.01, 0.1, 1, 10, and 100 µg/mL, and incubated for 24 h, or 0.1, 1, 10, 100, and 1000 µg/mL and incubated for 72 h. After exposure to nanoparticles for 24 or 72 h, the cultured HSF cells were fixed and washed with distilled water, and then, SRB solution (0.4% w/v) was added and the cells were incubated for 10 min at room temperature in a dark place. All plates were washed with acetic acid (1%) and left overnight to dry. The protein-bound SRB stain was then dissolved and the absorbance measured using a BMG LABTECH®-FLUO Star Omega microplate reader (Ortenberg, Germany) at 540 nm.

### Treatment Schedule

HSF cells were cultured under the proper conditions and divided into control and treated cells. Control cells were treated with an equal volume of the vehicle (DMSO; final concentration, ≤ 0.1%), while treated cells were treated with the determined IC50 of CaTiO_3_-NPs or Er_2_O_3_-NPs. All HSF cells were left for 72 h after treatment and harvested by trypsinization and centrifugation. Cells were then washed twice with ice-cold PBS and used for different molecular assays. Triplicate was done for each treatment.

### Genomic Stability

Alkaline comet assay was done to study the effect of CaTiO_3_-NPs and Er_2_O_3_-NPs separately on genomic DNA integrity in HSF cells [19, 20]. Briefly, a mixture of cell suspensions and low melting agarose was dispersed on a clean slide covered with a layer of normal melting agarose and left to dry. Slides were then incubated in a cold lysis buffer, electrophoresed, and finally immersed in neutral Tris buffer. Slides were immersed in cold absolute ethanol for permanent preparation. Immediately before examination, slides are stained with ethidium bromide, and photographed using an epi-fluorescent microscope at magnification 200 × , and fifty comet nuclei were analyzed using Comet Score™ software for each sample.

### Production of Intracellular ROS

The influence of CaTiO_3_-NPs and Er_2_O_3_-NPs separately on the production level of intracellular reactive oxygen species (ROS) in HSF cells was also studied based on the formation of a fluorescent dichlorofluorescein complex resulting from the reaction of a 2,7-dichlorofluorescein diacetate dye with intracellular ROS [21]. After culturing, HSF cells were washed with phosphate-buffered saline (PBS), and 2,7-dichlorofluorescein diacetate dye was added and left for 30 min in the dark. The mixture of cells and dye was then dispersed on a clean slide and the emitted fluorescent light was examined using an epi-fluorescent microscope at 200 × magnification.

### Measurement of Apoptotic Gene Expression Level

The mRNA expression levels of p53, Bax, and Bcl2 genes were measured using real-time polymerase chain reaction (RT-PCR) in all HSF cells after 72 h of treatment. Total RNA was extracted using the GeneJET RNA Purification Kit (Thermo Scientific, USA) and then transcribed reversely into complementary DNA (cDNA) using the Revert Aid First Strand cDNA Synthesis Kit (Thermo Scientific, USA). To measure mRNA expression levels, the p53, Bax, and Bcl2 genes were amplified using the 7500 Fast system (Applied Biosystem 7500, Clinilab, Egypt). The primers previously designed by [22, 23] listed in Table 1 were used for RT-PCR amplification and the comparative *Ct* (DD*Ct*) method was undertaken to quantify the mRNA expression levels of amplified genes. Data of RT-PCR were standardized using housekeeping GAPDH gene expression and the results were expressed as mean ± SD.

**Table 1:** Sequences of the used primers in RT-PCR

Gene	Strand	Primer’s sequences
GAPDH	Forward	5ʹ-GAAGGTGAAGGTCGGAGTCA-3ʹ
	Reverse	5ʹ-GAAGATGGTGATGGGATTTC-3ʹ
BAX	Forward	5ʹ-CCGCCGTGGACACAGAC-3ʹ
	Reverse	5ʹ-CAGAAAACATGTCAGCTGCCA-3ʹ
BCL-2	Forward	5ʹ-TCCGATCAGGAAGGCTAGAGT-3ʹ
	Reverse	5ʹ-TCGGTCTCCTAAAAGCAGGC-3ʹ
P53	Forward	5ʹ-CAGCCAAGTCTGTGACTTGCACGTAC-3ʹ
	Reverse	5ʹ-CTATGTCGAAAAGTGTTTCTGTCATC-3ʹ

### Statistical Analysis

All results of the present study are expressed as mean ± standard deviation (SD) and were analyzed using the Statistical Package for the Social Sciences (SPSS) (version 20) at the significance level *p* < 0.05. The Student *t*-test was used to compare between the untreated and treated cancer MCF-7 and normal HSF cells.

## Results

### Characterization of Nanoparticles

**Fig. 1:**
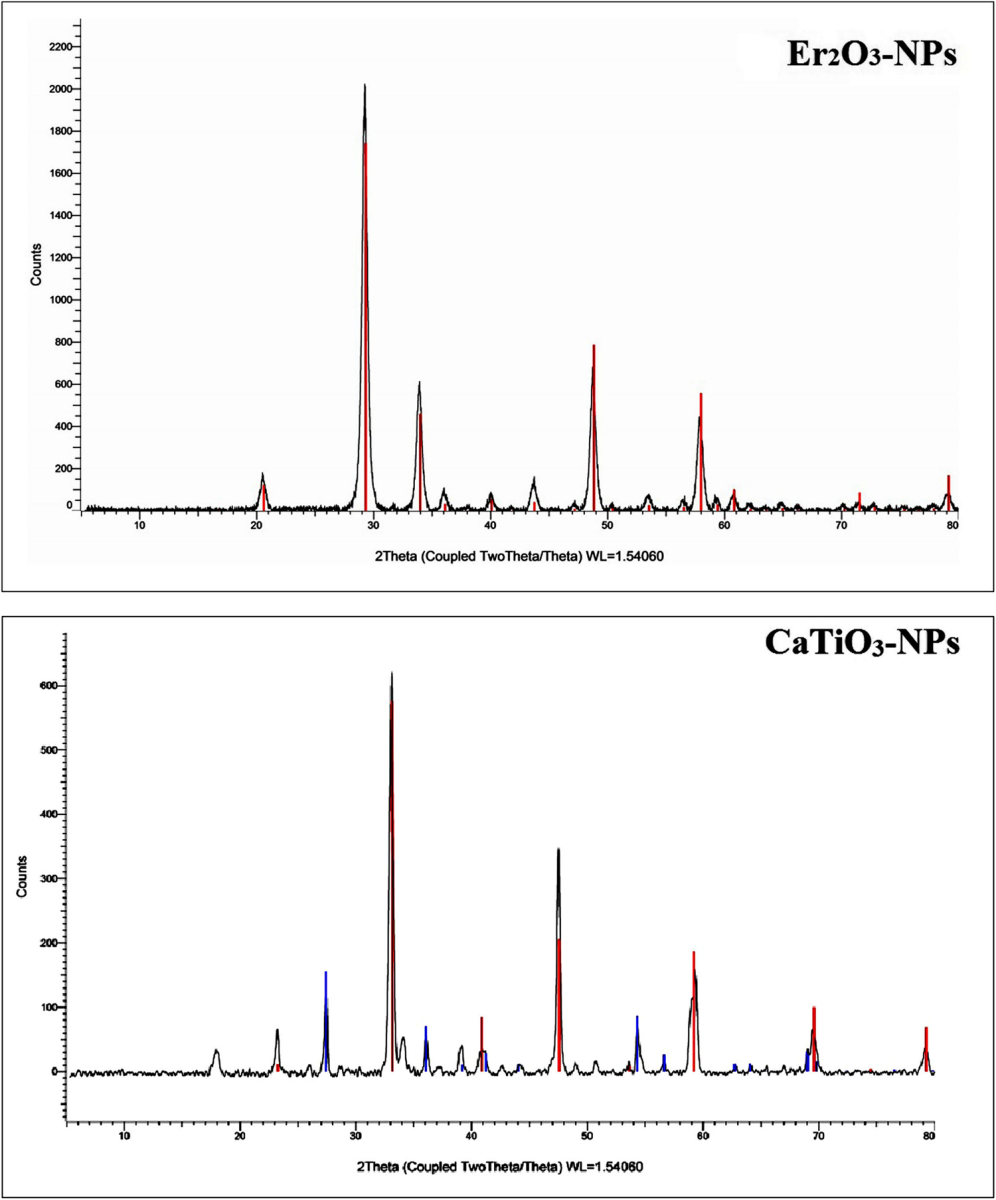
XRD pattern of erbium oxide (Er_2_O_3_) and calcium titanate (CaTiO_3_) nanoparticles (NPs)

As shown in Fig. 1, XRD analysis revealed the purity of the purchased CaTiO_3_-NP powders by the appearance of characteristic peaks for CaTiO3-NPs at theta angles of 32.7°, 47.1°, 58.9°, and 69°. TEM imaging showed that CaTiO_3_-NPs are well dispersed with a spherical shape and a mean size of 88.79 ± 22.32 nm (Fig. 3).

**Fig. 2:**
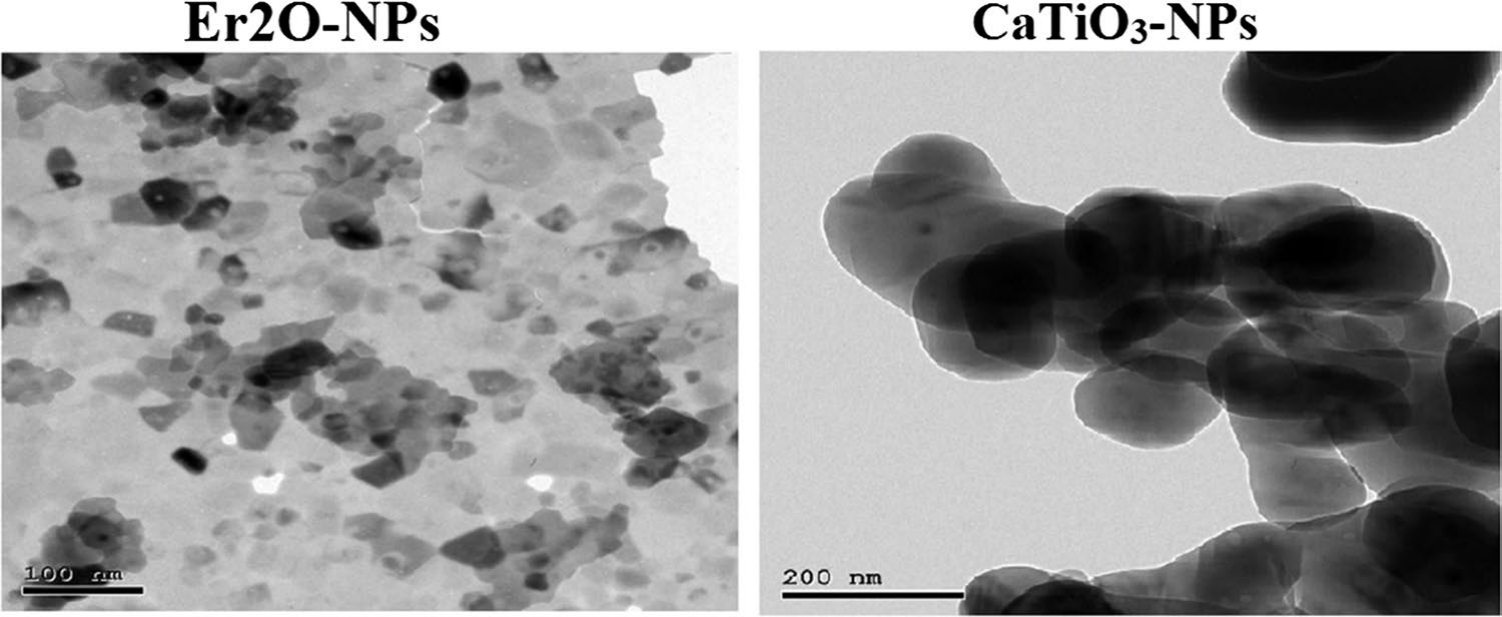
TEM imaging of erbium oxide (Er_2_O_3_) and calcium titanate (CaTiO_3_) nanoparticles (NPs)

The XRD pattern also confirmed the purity of the used erbium oxide nano-powders by the appearance of distinctive bands for Er_2_O_3_-NPs at the diffraction angles of 29.33°, 33.99°, 48.84°, and 58.00° as shown in Fig. 1. Imaging of the ultra-sonicated nanoparticles using TEM demonstrated the cubic shape and well dispersion of Er_2_O_3_-NPs with an average particle size of 50 ± 3.55 nm (Fig. 2).

### Cell Viability

**Fig. 3:**
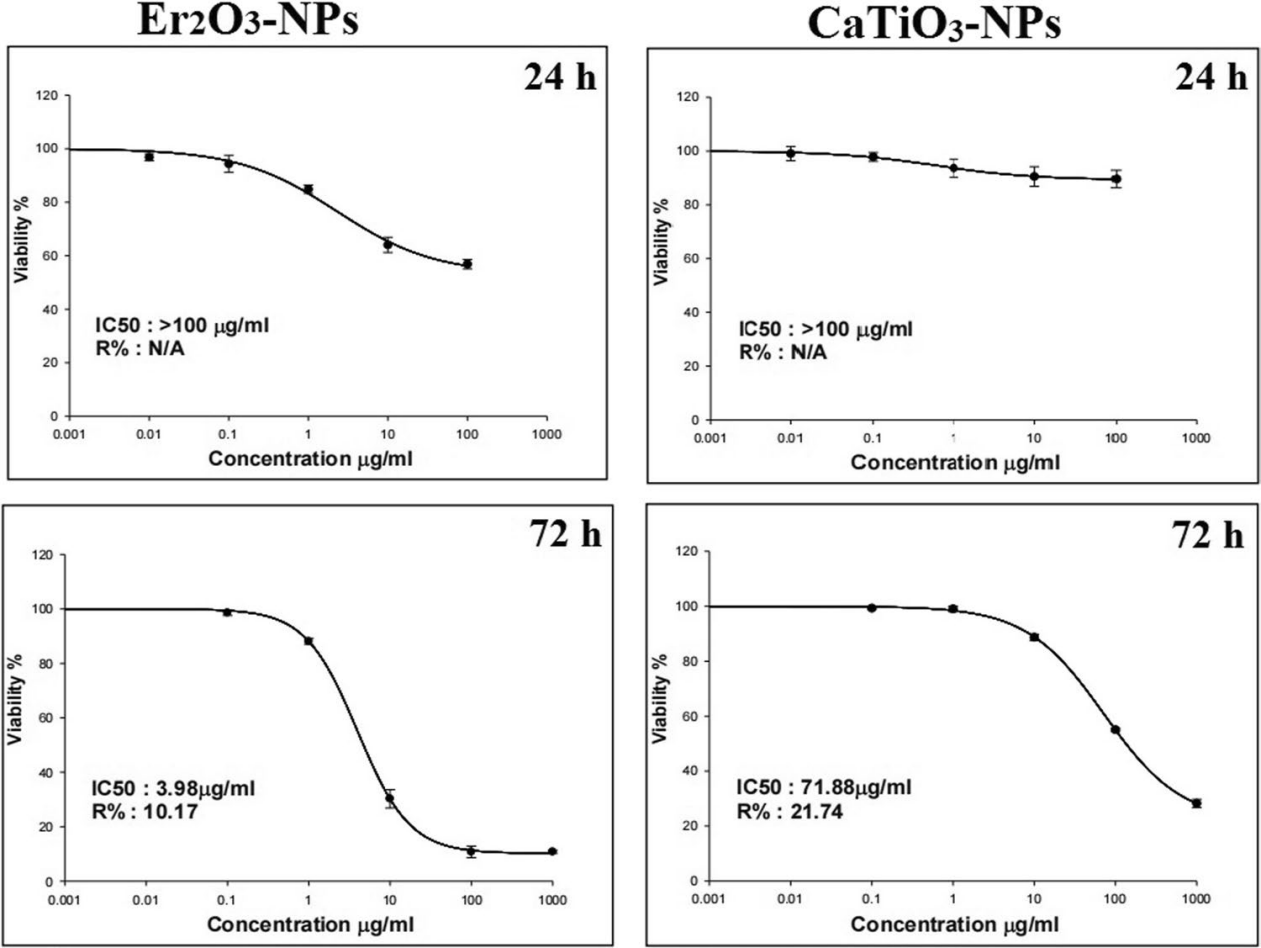
Cytotoxicity assay and viability of normal HSF cells after treatment with different concentrations of Er_2_O_3_-NPs or CaTiO_3_-NPs for 24 h and 72 h

The results of the SRB assay showed that treatment of normal HSF with CaTiO3-NP or Er_2_O_3_-NP different concentrations (0.01, 0.1, 1, 10, or 100 µg/mL) for 24 h did not affect cell viability and slight cell death was observed in cells treated with CaTiO_3_-NP or Er_2_O_3_-NP concentration greater than 10 µg/mL; thereby, the half maximal inhibitory concentration (IC50) at 24 h of CaTiO_3_-NPs or Er_2_O_3_-NPs was greater than 100 µg/mL in normal HSF (Fig. 3).

On the contrary, treatment of HSF cells with CaTiO_3_-NP or Er_2_O_3_-NP five different concentrations, i.e., 0.1, 1, 10, 100, or 1000 µg/mL, for 72 h significantly decreased cell viability in a concentration-dependent manner and the IC50 value at 72 h for CaTiO_3_-NPs was 71.88 µg/mL and for Er_2_O_3_-NPs was 3.98 µg/mL (Fig. 3).

### Genomic Instability

**Fig. 4:**
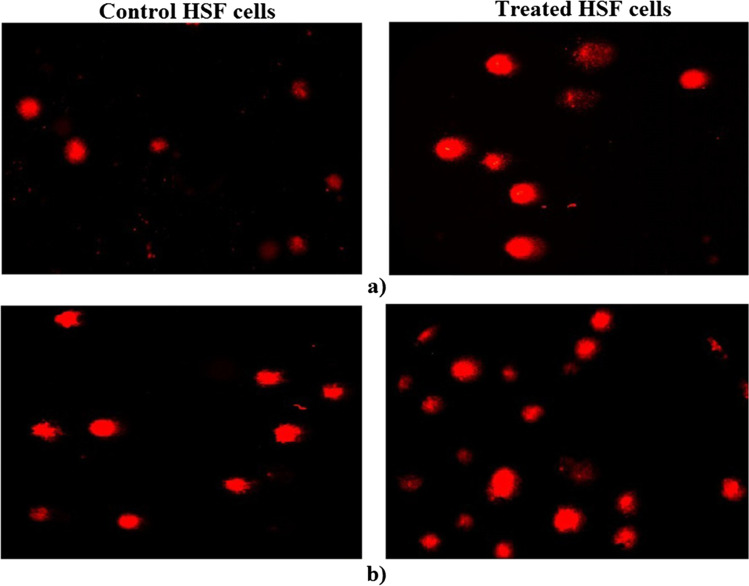
Examples for the scored comet nuclei in the control and treated HSF cells with IC50 of (**a**) Er_2_O_3_-NPs or (**b**) CaTiO_3_-NPs for 72 h

As shown in Table 2, no significant changes were observed in the measured DNA damage parameters: tail length, %DNA in tail, and tail moment after 72 h of normal HSF cell exposure to CaTiO_3_-NPs’ IC50 (71.88 µg/mL) or Er_2_O_3_-NPs’ IC50 (3.98 µg/mL) compared to the measure values in the untreated HSF cells. Examples for the examined and scored comet nuclei in the control and treated HSF cells are shown in Fig. 4.

**Table 2:** Tail length, %DNA in tail, and tail moment in the control and treated HSF cells with IC50 of Er_2_O_3_-NPs or CaTiO_3_-NPs for 72 h

Cell line	Treatment	Tail length	%DNA in tail	Tail moment
Er_2_O_3_-NPs	Control	5.15 ± 0.73	33.07 ± 6.50	1.93 ± 0.69
	Treated	4.83 ± 0.96	34.41 ± 7.85	1.95 ± 0.84
CaTiO_3_-NPs	Control	5.11 ± 0.77	33.88 ± 5.99	1.90 ± 0.70
	Treated	4.34 ± 0.45	32.84 ± 2.09	1.77 ± 0.41

Results are expressed as mean ± SD

### Intracellular ROS Production

**Fig. 5:**
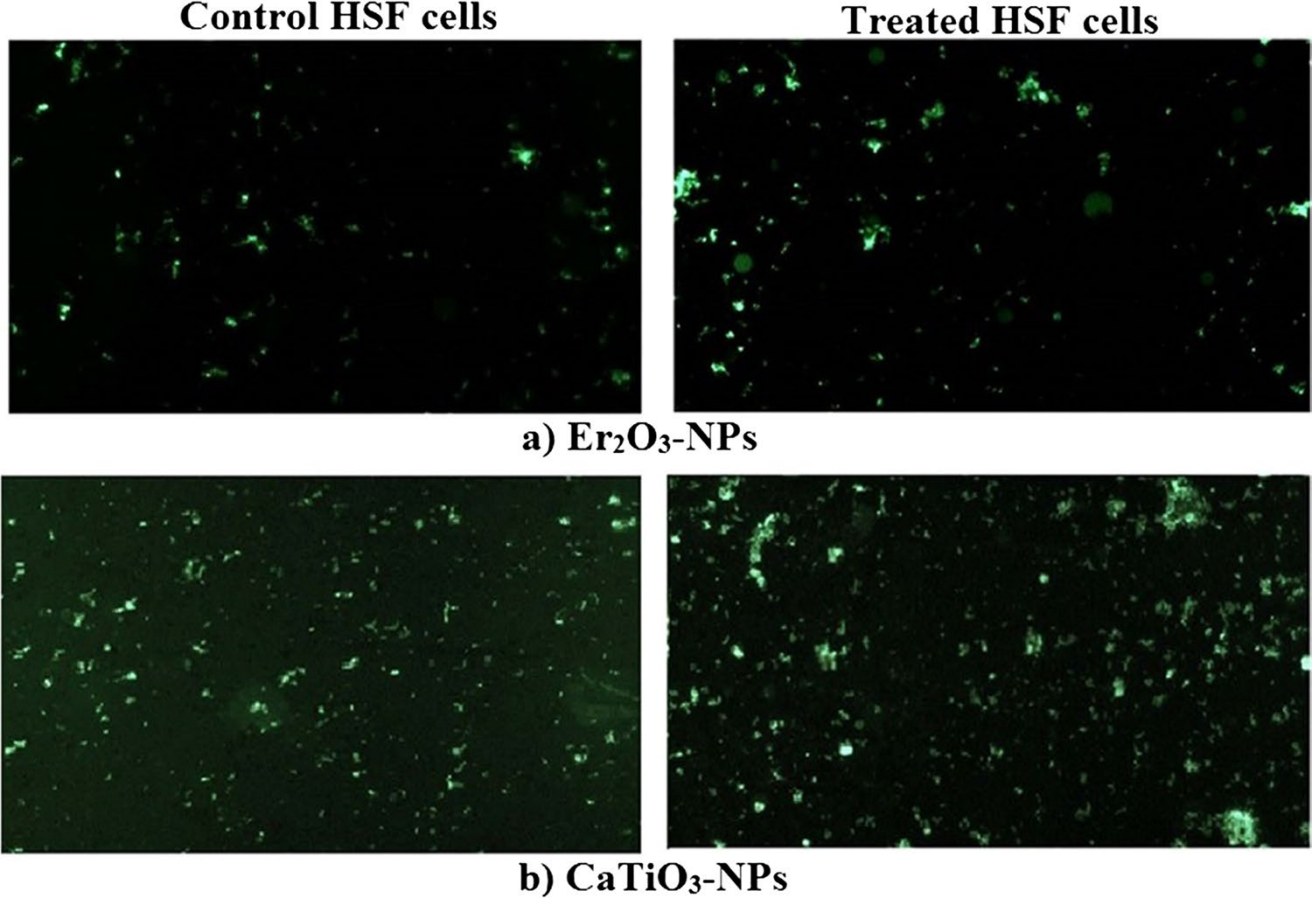
Intracellular ROS level in the control and treated HSF cells with IC50 of (**a**) Er_2_O_3_-NPs or (**b**) CaTiO_3_-NPs for 72 h

As shown in Fig. 5, treatment of HSF cells with 71.88 µg/mL (IC50) of CaTiO_3_-NPs or 3.98 µg/mL (IC50) of Er_2_O_3_-NPs for 72 h caused non-observable changes in the intracellular level of ROS generation as compared to the intracellular ROS level in the untreated control HSF cells (Fig. 5).

### Expression of Apoptotic Genes

As seen in Table 3, treatment of HSF cells with CaTiO_3_-NPs’ IC50 (71.82 µg/mL) or Er_2_O_3_-NPs’ IC50 for 72 h caused no marked changes in the mRNA expression levels of the p53, Bax, and Bcl2 genes compared to their expression levels in the untreated HSF cells.

**Table 3:** Expression levels of p53, Bax, and Bcl2 genes in the control and treated HSF cells with IC50 of Er_2_O_3_-NPs or CaTiO_3_-NPs for 72 h

Cell line	Treatment	P53	Bax	Bcl2
Er_2_O_3_-NPs	Control	1.00 ± 0.00	1.00 ± 0.00	1.00 ± 0.00
	Treated	1.24 ± 0.08	1.13 ± 0.06	1.02 ± 0.07
CaTiO_3_-NPs	Control	1.00 ± 0.00	1.00 ± 0.00	1.00 ± 0.00
	Treated	1.35 ± 0.41	1.19 ± 0.14	0.98 ± 0.11

Results are expressed as mean ± SD

## Discussion

Nowadays, the astonishingly rapid growth in the uses and applications of nanoparticles increases their environmental release and human exposure mainly in the workplace through skin contact. However, the dermal cytotoxic and genotoxic effects of CaTiO_3_-NPs or Er_2_O_3_-NPs separately have been poorly studied. Therefore, the current study estimated the effect of treatment with CaTiO_3_-NPs or Er_2_O_3_-NPs on the viability and integrity of the genomic DNA of normal HSF cells.

In the cytotoxicity test, HSF cells were treated with different concentrations of 0.01, 0.1, 1, 10, and 100 µg/mL for 24 h and 0.1, 1, 10, 100, and 1000 µg/mL for 72 h because this study was primarily designed to estimate the cytotoxic and genotoxic effects of sudden and prolonged exposure to CaTiO_3_-NPs or Er_2_O_3_-NPs. Therefore, normal HSF cells were exposed to a maximum concentration of 100 µg/mL of these nanoparticles for 24 h for a sudden momentary exposure test and also HSF cells were treated with tenfold (1000 µg/mL) for 72 h to study the effects of prolonged cumulative exposure to CaTiO_3_-NPs or Er_2_O_3_-NPs.

The results obtained from the SRB cytotoxicity assay demonstrated that CaTiO_3_-NPs or Er_2_O_3_-NPs induced time- and concentration-dependent cytotoxic effects since treatment with CaTiO_3_-NPs or Er_2_O_3_-NPs for 24 h was safe and caused non-observable changes in the viability of normal HSF cells. Meanwhile, prolonged exposure to CaTiO_3_-NPs or Er_2_O_3_-NPs for 72 h was cytotoxic and caused normal HSF cell death in a concentration-dependent manner. The cytotoxicity demonstrated in this study after prolonged exposure of HSF cells to CaTiO_3_-NPs supported the previously demonstrated cytotoxicity of CaTiO_3_ in previous studies [24, 25].

To further understand the toxic effects induced by CaTiO_3_-NPs or Er_2_O_3_-NPs, the levels of DNA damage and intracellular ROS generation as well as the expression levels of apoptotic genes were studied in normal HSF cells exposed to IC50 of CaTiO_3_-NPs or Er_2_O_3_-NPs for 72 h.

Highly reactive ROS molecules are naturally produced within cells during various metabolic processes. However, increases in intracellular ROS generations lead to an imbalance between oxidants and antioxidants within cells and damage cellular macromolecules: lipids, proteins, carbohydrates, and even DNA [26]. Overproduction of ROS within cells is one of the acceptable mechanisms for cell death mediated by several nanoparticles such as copper oxide, bismuth oxide, silver, and cobalt oxide nanoparticles [27, 28]. However, our finding of non-remarkable changes in the ROS level within the treated HSF cells demonstrated that prolonged treatment of HSF cells with IC50/72 h of CaTiO_3_-NPs or Er_2_O_3_-NPs did not alter the intracellular oxidant/antioxidant balance.

The alkaline comet assay is a highly sensitive and accurate cytogenetic and molecular genetic technique used in the detection of both single- and double-stranded DNA breaks [19]. As a result, the alkaline comet assay results indicated that CaTiO_3_-NPs and Er_2_O_3_-NPs are non-genotoxic as manifested by the non-observable changes in comet parameters: tail length, %DNA in tail, and tail moment measured after 72 h of normal HSF cell exposure to IC50/72 h of CaTiO_3_-NPs or Er_2_O_3_-NPs as compared to their values in the untreated HSF cells. These results are consistent with previous studies showing that cytotoxic nanoparticles are not necessary to be genotoxic because gold and silver nanoparticles, for example, are non-genotoxic even though they have shown cytotoxicity to human cell lines [29, 30].

Similarly, treatment of HSF cells with IC50/72 h of CaTiO_3_-NPs or Er_2_O_3_-NPs did not cause significant changes in the expression levels of apoptotic (p53 and Bax) and anti-apoptotic (Bcl2) genes. As a consequence, the safe and non-genotoxic effects of CaTiO_3_-NPs and Er_2_O_3_-NPs on normal HSF cells observed in this study may be due to genomic stability and regulation of various DNA repair mechanisms that enable normal HSF cells to maintain genomic DNA and cell integrity [12, 31]. Meanwhile, the cell death noticed after prolonged treatment of HSF cells with CaTiO_3_-NPs or Er_2_O_3_-NPs for 72 h may result from the release of a high amount of metal ions that directly attack cell membrane causing loss of its selective permeability and cell death [32].

## Conclusion

Based on the previously discussed data, although CaTiO_3_-NPs and Er_2_O_3_-NPs caused time- and concentration-dependent cytotoxicity toward normal HSF cells, prolonged treatment with IC50/72 h of CaTiO_3_-NPs or Er_2_O_3_-NPs for 72 h was safe and non-genotoxic as the genomic DNA integrity, ROS generation level, and apoptotic genes’ expression were non-significantly changed in treated normal HSF cells. Further studies on other cell lines and animal models in vivo are thus recommended to further understand the toxicological and biological effects of CaTiO_3_-NPs and Er_2_O_3_-NPs.

## Data Availability

The datasets used and/or analyzed during the current study are available from the corresponding author on reasonable request.
